# Bioluminescence-Based Energy Transfer Using Semiconductor Quantum Dots as Acceptors

**DOI:** 10.3390/s20102909

**Published:** 2020-05-21

**Authors:** Anirban Samanta, Igor L. Medintz

**Affiliations:** 1 Ramakrishna Mission Vidyamandira, Belur Math, Howrah 711202, India; anirbansamanta@vidyamandira.ac.in; 2 Center for Bio/Molecular Science and Engineering, Code 6900, U.S. Naval Research Laboratory, Washington, DC 20375, USA

**Keywords:** quantum dot, bioluminescence, FRET, luciferase, BRET, biosensing

## Abstract

Bioluminescence resonance energy transfer (BRET) is the non-radiative transfer of energy from a bioluminescent protein donor to a fluorophore acceptor. It shares all the formalism of Förster resonance energy transfer (FRET) but differs in one key aspect: that the excited donor here is produced by biochemical means and not by an external illumination. Often the choice of BRET source is the bioluminescent protein *Renilla* luciferase, which catalyzes the oxidation of a substrate, typically coelenterazine, producing an oxidized product in its electronic excited state that, in turn, couples with a proximal fluorophore resulting in a fluorescence emission from the acceptor. The acceptors pertinent to this discussion are semiconductor quantum dots (QDs), which offer some unrivalled photophysical properties. Amongst other advantages, the QD’s large Stokes shift is particularly advantageous as it allows easy and accurate deconstruction of acceptor signal, which is difficult to attain using organic dyes or fluorescent proteins. QD-BRET systems are gaining popularity in non-invasive bioimaging and as probes for biosensing as they don’t require external optical illumination, which dramatically improves the signal-to-noise ratio by avoiding background auto-fluorescence. Despite the additional advantages such systems offer, there are challenges lying ahead that need to be addressed before they are utilized for translational types of research.

## 1. Introduction

The ‘cold’ flashing light of fireflies has fascinated and intrigued people for centuries. It still delights us, except that now we know that it is the result of an enzyme catalyzed chemical reaction, which dissipates a minimum possible amount of heat. Besides fireflies, bioluminescence can be found in a variety of other life forms ranging from unicellular bacteria to numerous more complex terrestrial and aquatic organisms. While the mechanism of converting chemical energy into light is fundamentally the same among different species, the chemical nature of the substrates varies widely and the genes responsible for the catalytic enzymes show no homology to each other, indicating that these luminous systems probably have evolved independently [1]. Irrespective of their origin, the substrate for the oxidation is generically called luciferin and the enzyme is named luciferase.

**Figure 1:**
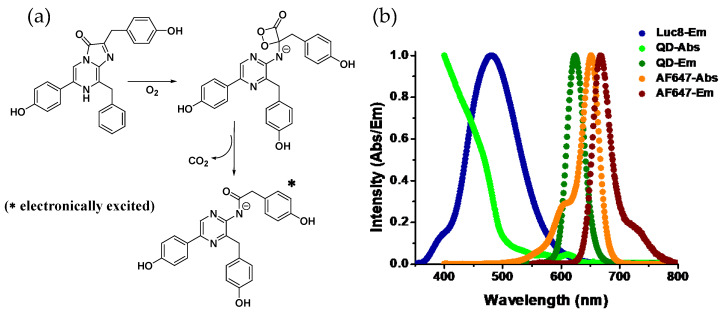
(**a**) Reaction mechanism of Renilla luciferase: catalyzed oxidation of coelenterazine to coelenteramide involving a typical strained four-membered cyclic peroxide intermediate with high energy content. The decomposition of the peroxide is exothermic resulting in electronic excitation of the product with the ability to emit light upon relaxation. (**b**) Plots displaying the normalized emission spectrum of Luc and both absorption and emission profiles of quantum dots (QD)-655 and the organic dye Alexa Fluor-647. While both the QD and the AF647 emit in a similar spectral range, due to the broad absorption spectrum of the QD and therefore large spectral overlap with the emission spectrum of Luc, energy transfer is far more favorable to the QD than to the dye. This is facilitated by the QD’s extinction coefficient of ~7,500,000 M^−1^cm^−1^ at 450 nm as compared to that of AF647 (260,000 M^−1^cm^−1^ at 647 nm) along with their respective quantum yields of 50% and 33%, respectively.

A common feature among luciferases is that they all are enzymatic oxygenases. Using molecular O_2_, the multistep oxidation reactions they catalyze usually occur through a peroxy (-O-O-) intermediate (Figure 1a). The role of a peroxy intermediate is crucial as it provides a route for thermal activation of the ground state molecule to the electronically excited product that is capable of emitting light [2,3]. For example, *Renilla* luciferase, found in the sea pansy *Renilla reniformis*, assists oxidizing of coelenterazine, the luciferin, to produce coelenteramide and CO_2_ with concomitant emission of blue light (λmax~480 nm). Photons of that wavelength corresponds to ~60 Kcal/mole of energy, which is nearly eight times higher than the hydrolysis enthalpy of one mole of ATP molecule to ADP. So it is apparent that photon-generating reactions have to be highly exergonic. It is proposed that a stereochemically strained four-membered energy rich cyclic peroxide forms during the course of the reaction which decomposes to produce stable CO_2_ and a carbonyl (C=O) compound while releasing a large amount of energy, which thermochemical calculations suggest is about 70–90 Kcal/mole, sufficient for the electronic excitation of the carbonyl product—coelenteramide in this case [4,5]. Depending on the chemical nature of the substrate and the physiological condition they are in, emission wavelengths can vary. Luciferases also play a key role here. Specific regions in the protein have been identified where amino acid substitutions seem to cause significant shift in the emission wavelength, and this is attributed to the change in torsion a substrate/product experiences in the binding pocket of the enzyme that modifies the electronic states of the enzyme-bound product (for the sake of brevity, henceforth we’ll call it Luc) [6,7].

Emission spectra of Luc may simply look like the externally illuminated fluorescence spectra of the oxidized products, but often in vivo it appears to be red-shifted. This happens in the presence of an accessory fluorescent protein (FP), such as green fluorescent protein (GFP), when located in close enough proximity to allow some transfer of energy from Luc to the FP, which results in emission of light from the latter at a higher wavelength [8]. The mechanism of this energy transfer is called bioluminescence resonance energy transfer or BRET. BRET shares all the formalism of Förster resonance energy transfer (FRET), i.e., the transfer does not involve emission and re-absorption of photon and is the result of a long-range coupling between the transition-dipoles of the two participating fluorophores [9]. Akin to FRET, the rate of energy transfer in BRET also displays an inverse sixth power distance dependency. Besides distance, other factors such as the spectral overlap between donor luminescence and acceptor absorption spectra and mutual orientation of the relevant dipoles influence the energy transfer efficiency in the same way as FRET does [10]. Despite the similarities, BRET offers some special advantages that FRET cannot afford, primarily due to the chemical nature of excitation in BRET. In FRET, for example, when the donor is excited via external illumination, molecules other than the target fluorophores abundantly present in a biological sample may also get excited and faintly fluoresce but collectively this auto-fluorescence may substantially reduce the signal-to-noise ratio in a FRET experiment. The same light source can also excite the acceptor fluorophore, albeit weakly, complicating the subsequent spectral deconvolution and data analysis. Moreover, strong laser light typically used in FRET studies may cause damage to the molecular framework of a fluorophore, rendering them incapable of fluorescing further if a second round of excitation is required. Obviating the need for external light, BRET is not plagued by these issues. Naturally, it has turned out to be a powerful tool for evaluating macromolecular interactions and a basis for developing new classes of biosensor, probes, anti-cancer agents, and even light-harvesting and directing nanodevices [11,12].

While the acceptors in BRET have traditionally been FPs, chemically conjugated organic fluorophore dyes have also served the same purpose, [13] but the BRET acceptor pertinent to our discussion that of an emissive inorganic nanoparticle—namely luminescent semiconductor quantum dots (QDs). QDs are nanometer-sized colloidal nanocrystalline particles that display unique quantum confined opto-electronic properties. As a fluorophore, QDs offer some unrivalled photophysical properties that include broad absorption spectra in association with a narrow and symmetric photoluminescence (PL) profile—the latter being tunable via changing of the chemical composition and/or size of the particles (Figure 1b). They also have high quantum yields and display remarkable resistance to photo and chemical degradation. While use of QDs as FRET donors is ubiquitous, their role as an acceptor is limited by their long PL lifetime (5–50 nanoseconds). QDs display several unique physicochemical properties that help augment their role as a BRET acceptor, these are outlined in Table 1. They are qualified as an acceptor only when the associated donor has a comparable radiative lifetime, which is observed in lanthanide phosphorescence or processes like bio or chemiluminescence but not among most of the available conventional organic fluorophore dyes [14,15,16,17]. Their broad absorption spectra allow them to be excited at a wavelength far away from the discrete PL, contributing to their large effective Stokes shift. This is especially advantageous for BRET as virtually all bioluminescent proteins can be used for QD excitation and, in addition, it simplifies the task of spectral separation of acceptor emission from that of the donor—something that tends to be far more challenging with other BRET acceptors like dyes (Figure 1b). In addition, the large, nontrivial surface area of the QDs allows stoichiometric attachment of multiple acceptor fluorophores around them in a relatively centrosymmetric manner. The latter, in turn, increases the energy transfer efficiency in a controllable manner by proportionally increasing the effective acceptor absorption cross section. Cumulatively, these properties have spurred significant interest in developing QD–Luc conjugates, which beyond its current application as bioimaging and sensing probes can potentially have broader impact on different facets of scientific research requiring challenging fluorescent formats.

## 2. System Design and Ligation Strategy

The emission maxima of natural and mutant luciferase variants typically span ~460 to ~620 nm. Amongst varieties of natural and mutant variants, Renilla luciferase (RLuc8) and Firefly luciferase (FLuc) are the two widely employed proteins for BRET studies, having emission maxima around 480 (blue-green) and 560 nm (yellow-green), respectively. RLuc8 differs from its native form by eight introduced site-specific mutations and displays brighter luminescence and strong resistance against deactivation in serum [18]. RLuc8 uses coelenterazine and O_2_ while FLuc, besides requiring the substrate D-luciferin and O_2_, also needs ATP and Mg^2+^ as cofactors to generate luminescence. The ATP dependence of the latter is often viewed as a drawback for in vivo use as the concentration of ATP in serum is fairly low [19]. Besides these two, newly introduced NanoLuc is another mutant luciferase that has less frequently been fused with QDs. It has an emission maxima ~20 nm blue shifted from that of RLuc8 but displays luminescence that is nearly 150 times brighter [20]. While its brightness in conjunction with relatively long-lived luminescence, small size, and ATP independence are quite appealing, the substrate it uses, furimazine, is a coelenterazine derivative and currently not generically available—so cost can be a factor here. Nevertheless, a variety of QDs can ideally be coupled to them that can facilitate significant energy transfer-thanks to the broad absorption spectra of QDs that lead to substantial spectral overlap with the broad emission of the donor. The metric for BRET efficiency, formally called the BRET ratio, is expressed in terms of the ratio of integrated emission of the acceptor to that of the luciferase. To calculate the BRET ratio, the contribution of the acceptor and donor in a composite raw spectrum needs to be precisely evaluated. This is generally done by deconvoluting the spectrum and deconvolution is easy and accurate if the spectrum of the two are distinctly separated. BRET-sensitized QD emission above 640 nm can comfortably be resolved from the Luc PL, which probably explains the popularity of utilizing the commercially available QD655 (λmax, em = 655 nm, ThermoFisher) as the BRET acceptor. QD655 is a core-shell QD where the core, a CdSe nanocrystal, is encapsulated in a few nanometers thick shell of ZnS. Being a semiconductor nanocrystallite, CdSe core-only QDs themselves are capable of emitting upon irradiation, but a shell of wider band gap material such as ZnS improves their quantum yield by passivating surface defects making them look much brighter [21]. A shell at the same time helps to reduce toxicity by preventing Cd^2+^ leaching, which remains a longstanding concern for their in vivo use [22]. Cumulatively, these added advantages have made core-shell QDs more desirable over core-only QDs for bioimaging and biological assays, [23] with no exception when constructing QD–Luc BRET systems.

**Figure 2:**
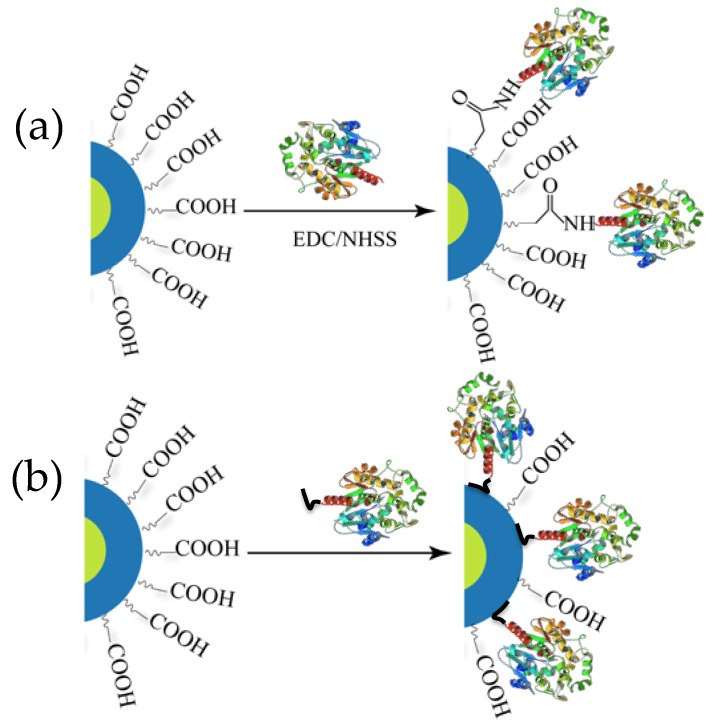
Schematic depicting two major ways of conjugating luciferase to QDs. (**a**) Chemical conjugation via 1-ethyl-3-(3-dimethulaminopropyl)carbodiimide (EDC) coupling chemistry. (**b**) Spontaneous self-assembly via His_6_ mediated metal affinity coordination where the imidazole side chain of His residues binds to the Zn^2+^ of the ZnS shell of the QD. His_6_ motifs are usually recombinantly inserted to one end of the enzyme for purification of Ni^2+^ chelate media.

QDs, whether the final hydrophilic version or the initial hydrophobic native product, are always coated with some kind of surface ligand. Without these surface ligands QDs cannot exist in a colloidal state but the ligands serve other important roles too [24]. Often the ligands on hydrophilic QDs display carboxylic acid groups that primarily serve two purposes: (i) they impart aqueous solubility (i.e., colloidal stability in water) necessary for biological application and (ii) they can display terminal functional groups for bioconjugating and displaying a variety of biomolecules on the QDs, including proteins, peptides, and DNA, to name a few [25]. The first report describing BRET between luciferase and a QD, and later other related reports, utilized surface carboxylic acid groups to ligate luciferase to the QD by exploiting EDC-based carbodiimide coupling chemistry (Figure 2a) [26]. EDC is the abbreviated name of the 1-ethyl-3-(3-dimethulaminopropyl)carbodiimide which is used in conjunction with NHS (N-hydroxysuccinimide) to activate carboxylic acid groups that react to primary amines to form amide bonds at a faster rate, yielding more product. However, primary amines and carboxylic acid groups are ubiquitous in proteins, so crosslinking and subsequent aggregation is a common issue. In addition to this, extensive purification is often required to remove excess reagents, unreacted proteins, and uncoupled QDs and this process also seems to have a detrimental impact on the PL quantum yield (QY) of the resulting construct [27].

The majority of reports concerning BRET from Luc to QD, however, involve a different type of bioconjugation chemistry based on metal affinity coordination (Figure 2b). The metal ion pertinent to our discussion is Zn^2+^, present on the ZnS shell of many core-shell QDs and which manifests high affinity for imidazole functionalities on the side chain of the amino acid histidine. Histidine (His) residues are quite common in proteins but the affinity of discrete His residues for Zn^2+^ is not strong enough to ensure a robust conjugation [28]. Contiguous histidines (His_n_, where n is typically 6) can be incorporated recombinantly into either terminus of proteins without affecting their original function. Presently, this is a routine process practiced in molecular biology labs for purifying protein using Ni-NTA columns where Ni^2+^ ions forms a chelated compound with resin–immobilized NTA and the His-tag [29]. The same concept has been successfully adopted for appending His-tags to luciferases and subsequently attaching them directly onto the Zn-rich QD surface [25]. The process requires only mixing of the two components and allowing the mixture to stand for 5–10 min, within which the assembly is believed to reach equilibrium. With a dissociation constant reportedly in the order of 10^−9^ M, the multidentate dative interaction is evidently much stronger and effectively gives rise to near-stoichiometric product with a narrow distribution at higher ratio [30,31,32,33]. This approach provides other benefits as it does not cross-link QDs and does not detrimentally affect their QY. Indeed, a slight increase in the QD PL intensity has been observed following conjugation, probably due to better surface passivation that is believed to reduce non-radiative electron-hole recombination events that arise due to dangling bonds on the QD surface [34].

## 3. Applications

### 3.1. Bioimaging

Most QD–Luc constructs have found applications in sensing and bioimaging. Luciferases themselves have been widely used to observe biomolecular processes in vivo in real time. The common practice there is to introduce the luciferase reporter gene into the cells of interest and allow the protein to be expressed. This is followed by exogenous addition of respective cell-permeable luciferins, allowing the bioluminescent cells to be seen and probed to obtain information about their trafficking, growth, or death using highly sensitive modern microscopes [35]. The same can be achieved with fluorescent protein, however, there are a number of issues that plague common fluorescence imaging techniques. First and foremost is the difficulty of incident light to penetrate and excite a fluorescent probe located deep inside a tissue due to the combined effect of absorption, scattering, and reflection of the light in the tissue; second is the scattering and absorption of emitted light elicited again by tissue; and third, which is applicable to thick as well as thin biological samples, is the autofluorescence from numerous naturally occurring biomolecules contributing to background noise [26,36,37,38]. Cumulatively all three ultimately reduce the signal-to-noise ratio in an acquired image or spectrum leading to an inconclusive result. Since the need for extrinsic illumination is eliminated in bioluminescence imaging (BLI), the question of paucity of exciting light inside deep tissue does not now arise, which improves imaging capabilities, especially in whole animals. This comes in conjunction with a much-improved signal-to-noise ratio due to the negligible presence of endogenous light, making BLI superior among other optical imaging modalities in terms of sensitivity. However, the emitted light in BLI does have to transmit through tissue and get absorbed by pigmented biomolecules therein, especially those photons from the blue-green region (450–550 nm) of the spectrum where Luc8 and FLuc typically emit [36]. Light beyond the wavelength of 650 nm, also known as near infrared or NIR light (650–1000 nm), is less absorbed by most of the tissues since it begins to overlap with what is referred to as the first tissue transparency window. There are numerous QDs reported in the literature [37], and some even are available commercially, that emit in the NIR window and these have already been used for deep tissue imaging [38]. Therefore conjugating luciferase to such QDs can solve the issue of blue light absorption if BRET occurs between the two. Therefore, using QD–Luc as an imaging probe, the microscope effectively sees sensitized QD NIR emission as well as attenuated emission from luciferase after transmission to QD and absorption by tissues on its way to reach the detector. The intensity ratio of the two emissions can be used instead of an error-prone absolute value of light intensity as a parameter to gauge the target biological process.

**Figure 3:**
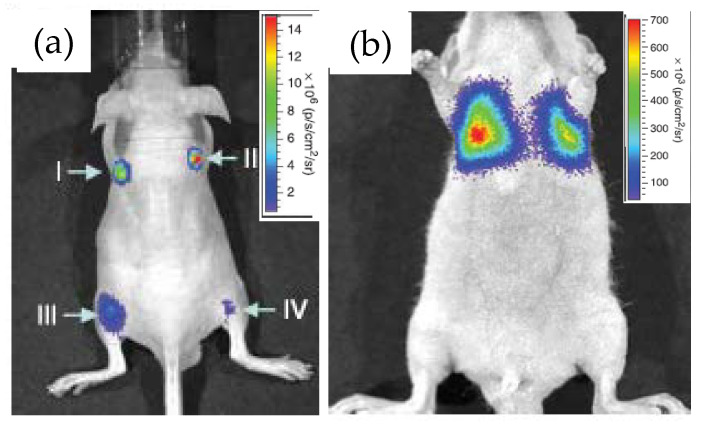
Images displaying application of the QD655-Luc system as an efficient in vivo imaging probe. (**a**) Strong BRET signal emanated from four different injection sites, subcutaneously (I and II) and intramuscularly (III and IV) in a live mouse. (**b**) BRET signal from QD655-Luc labeled C6 rat glioma cells that were injected in the tail vain but subsequently trafficked near the lungs. Reprinted with permission from Springer Nature [26], Copyright 2006.

Rao’s group at Stanford University first demonstrated the use of RLuc8 conjugated QDs as an in vivo bioimaging agent and coined the term “self-illuminating QDs” for obvious reason [26]. The conjugation was performed via the EDC coupling route and the resulting construct displayed decent stability in biological fluids such as serum and blood, as evidenced by a strong BRET between the two. When QD–RLuc8 was injected inside a nude mouse, subcutaneously at one site and intramuscularly in another, and coelenterazine was then injected in the tail-vein. Irrespective of the injection sites, strong detectable signals emanated in both cases from the BRET-sensitized QD with emission maxima at 655 nm (Figure 3a), the latter represent the commercial QD materials described above. In a control experiment, injection of RLuc8 alone gave rise to a much weaker signal from the deep tissue due to absorption and scattering of the short-wavelength bioluminescence. They also demonstrated the potential application of these constructs as a multiplexed imaging unit by conjugating RLuc8 to three different QDs with emission maxima at 655, 705 and 800 nm. BRET sensitized emissions from all of the conjugates, separately as well in a mixture, were clearly observed at four different injected sites in the same mouse. The only issue was some spectral cross talk between QD655 and QD700, and QD700 and QD800, which should be resolved with the continuing development of finer optical filters. The prospect of the conjugates for cell labeling and subsequent monitoring of their translocation inside an animal was also explored. The approach, however, necessitated the QD–Luc particles to be functionalized with short pieces of a polycationic peptide, again via EDC coupling, to facilitate their cell uptake [39,40,41]. The particles taken up inside C6-glioma cells did display substantial BRET-sensitized emission from the QDs, which did not get attenuated when the injected cells were inside the mouse and accumulated near its lungs (Figure 3b). For comparison purposes, fluorescence spectral imaging was performed in each case by exciting the QDs with external irradiation. Although relatively strong fluorescence signal was observed from superficial depths, the signal, however, was suboptimal from deeper tissue.

This success in using QD–Luc BRET system as an efficient bioimaging agent opened up other avenues of research as well, for example, in protease sensing, photodynamic therapy, and imaging specific regions of the body with a pivotal physiological function such as lymph nodes. Lymph nodes have key roles in maintaining proper immune response and in cancer metastasis and therefore their visualization has important clinical implications. Researchers have successfully employed the QD–Luc BRET system for lymphatic imaging with extremely low background signal in mice [42]. They injected commercially available QD–RLuc8 conjugates to various lymphatic sites in mice and clearly observed the lymph nodes under an optical microscope even after an hour of coelenterazine administration. They further envisioned, based on preliminary results, that this technology could be applied for target specific imaging of malignant tumors if the nanocomposite is further functionalized with cancer recognizing moieties like a monoclonal antibody. In 2016, Kamkaew and colleagues reported on similar aspect of target specific imaging using a novel luciferase, named nano luciferase or NLuc—a mutant version of the one originally found in a specific deep-sea shrimp [43]. The prefix ‘nano’ originates from their relatively small size ~19 kD, compared to RLuc and FLuc having sizes 36 and 65 kD, respectively. EDC coupling chemistry was exploited to conjugate NLuc and a target-binding unit to polymer coated CdSe/ZnS core-shell QDs with emission maxima at 705 nm, which falls in the NIR range and suffers minimal absorption by animal tissue. The targeting unit was a small cyclic peptide known to have strong affinity to αvβ3 cell surface receptors overexpressed in a majority of tumor cells. Significant BRET was observed both in vitro and in vivo, enabling visualization of specific lymph nodes even after two hours of substrate injection. The conjugates also displayed targeting capability as BRET-sensitized emission from the QDs were clearly visible from the expected sites within the tumor bearing mice.

Just as described in numerous reports QD surfaces have been modified with target recognizing biomolecules [44], however, the presence of luciferase on the QD offers a unique opportunity to append such moieties to the luciferase itself without affecting its intrinsic luminescence characteristics. In a recent paper, researchers have developed a target specific imaging probe involving QD and a recombinantly-expressed luciferase with a terminal His_6_ sequence for anchoring to the QD surface and Annexin V for the intended role of targeting [45]. Annexin V is an endogenous protein and a well-known probe for detecting apoptotic cells due to its strong binding affinity to cell membrane phospholipid phosphatidylserine that get exposed on the surface of a cell that is about to undergo apoptosis (or in other words programmed cell death). Cancer cells evade apoptosis, so visualization of apoptotic cells is important from a clinical perspective. The Annex V–Luc–QD conjugates displayed distinctive binding ability to breast cancer cells where apoptosis was induced by chemical means. The higher signal-to-noise ratio from the BRET-sensitized NIR QD emission compared to standard fluorescent dye labeled Annexin V probe again proved the potential of these conjugates as an optical imaging tool. The same research group applied a similar strategy for imaging of cancer cells by targeting the overexpression of a transmembrane protein called epidermal growth factor receptor (EGFR) which is commonly found in many cancer cells [46]. The targeting molecule was an EGFR specific antibody, which was linked to QD-bound Luc via an antibody binding domain genetically fused to Luc during its expression. While the detection sensitivity improved substantially compared to standard fluorescence imaging by exciting the QDs alone, this research also opened up possibilities of attaching a variety of other antibodies and implement the resulting constructs for various targeted imaging application.

### 3.2. Biosensing

**Figure 4:**
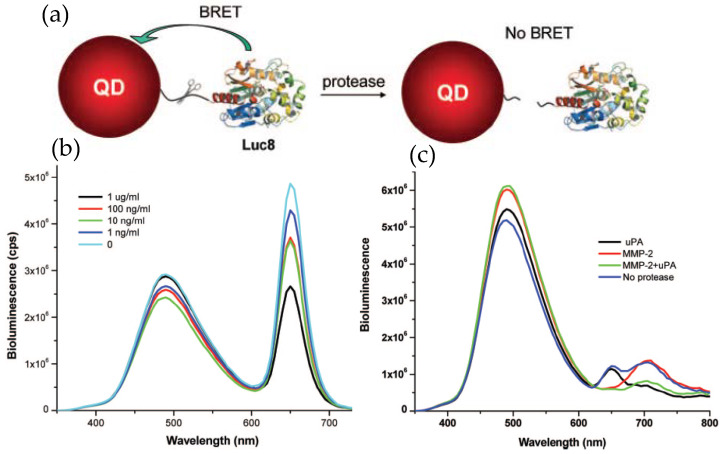
Application of QD–Luc conjugates as a protease sensor. (**a**) Schematic displaying the detection principle where the target protease cleaves its substrate peptide allowing the QD and Luc to diffuse away and concurrently reducing the BRET-sensitized QD emission. (**b**) Representative emission spectra displaying reduced QD emission with increased MMP-2 concentration. (**c**) Simultaneous detection of two different proteases, MMP-2 and urokinase (uPA), with two different probes, QD655-Luc and QD705-Luc, respectively. In the presence of specific protease, a drop in QD intensity at its respective peak was observed. Reprinted with permission from [48]. Copyright 2008 American Chemical Society.

The examples described above clearly imply the potential of QD–Luc system as a powerful imaging tool, the same may be true for their employment as a sensing probe. Rao’s group employed Luc8 coupled QDs for detecting proteolytic activity of the matrix metalloproteinases MMP-2 and MMP-7, which are believed to have vital roles in degrading extracellular proteins and seem to overexpress in various other pathological conditions [47]. The detecting principle was to modulate BRET efficiency by effecting a distance change between the donor and acceptor in the presence of the protease. This was accomplished by inserting a peptide sequence that could act as a substrate for the protease in question between the donor Luc8 and acceptor QD. Hydrolytic cleavage of the linker peptide by the protease separated the donor–acceptor, allowing them to diffuse away and concurrently resulting in a diminished BRET efficiency. The conjugate was prepared by genetically fusing the substrate peptide and a His_6_ sequence to the C-terminus of Luc8 and subsequently attaching it to the QD surface, as mediated via Ni^2+^ metal affinity. Elimination of the background fluorescence from naturally occurring fluorophores greatly improved the sensitivity with a detection limit down to a few nanograms per milliliter. The specificity of detection was also high enough to ensure simultaneous detection of two different proteases in complex biological fluid (Figure 4) [48]. In that case, two different proteases had to be linked to two different QDs, with emission maxima at 655 and 705 nm, via their respective substrate peptides.

It is not always necessary to conjugate luciferase to QDs. Signal transduction can also be turned on by the physical association of the two effected by noncovalent interaction of two different entities, a complex biomolecule such as DNA or an antibody or a simpler analyte conjugated separately to QDs and luciferase. However, such association can be disrupted by the presence of similar biomolecules or analytes that competitively bind to either QD or luciferase appended biomolecules, letting the donor/acceptor part ways and concurrently attenuating the BRET signal. This sort of competitive binding has been widely exploited for developing biosensors [49] and in the current context the same has been adopted for the detection of DNA and smaller molecules of special importance. As a proof of concept for detecting a target DNA, Cissel and coworkers conjugated a DNA strand identical to the target one to RLuc and its complementary strand to a NIR light emitting QD [50]. Hybridization of the two strands brought the RLuc and QD in close proximity, which resulted in a strong BRET-sensitized signal from the QD when exposed to coelenterazine. Progressive addition of the target DNA proportionally displaced the Luc labeled strand resulting in a decreased BRET-sensitized emission from the QD, the quantification of which allowed detection of DNA at a concentration as low as 20 nM. A similar strategy was followed for the detection of specific antibiotics, currently related to food safety issues due to their excessive use in livestock [51]. Enrofloxacin (ENR) is one such antibiotic that comes under the group of fluoroquinolone antibiotics and displays binding affinity to ScFv (abbreviation for single chain variable fragment, a fusion of the variable regions of IgG antibody), as do other fluoroquinolones. To detect ENR, QDs were conjugated to a different fluoroquinolone antibiotic namely norfloxacin (NOR) and RLuc was recombinantly fused to ScFv. Association of the two turned the BRET on, but presence of ENR turned the same off by preferentially binding to the ScFv, presumably at the same site, separating the NOR conjugated QD from the RLuc.

### 3.3. Therapeutics

**Figure 5:**
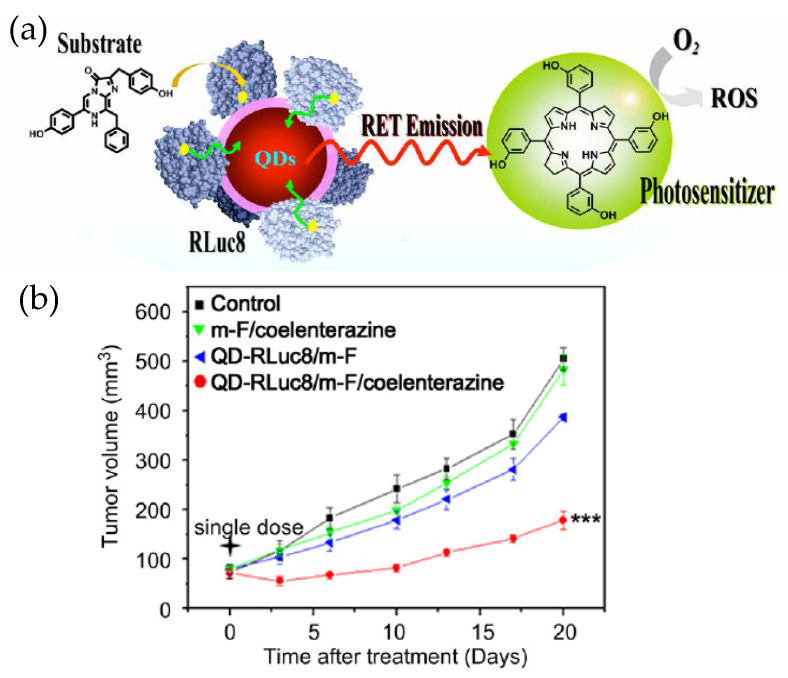
(**a**) Schematic depicting application of a QD–Luc BRET system for photodynamic therapy. The luciferase labeled QD and the micelle loaded photosensitizer encountered each other while they were being taken up by the cancer cells via endocytosis. The QD–Luc conjugates served as an endogenous light source for the photosensitizer to generate lethal reactive oxygen species (ROS). (**b**) Relative tumor growth curve showing the effectiveness of photodynamic therapy (PDT). The other traces are controls (m-F stands for micelle-Foscan). Reprinted with permission from [52]. Copyright 2013 Elsevier.

Besides being an imaging and analytical tool, QD–Luc BRET systems can have more direct impact on therapeutics as they have been used as an internal light source for photodynamic therapy (PDT) (Figure 5) [52]. PDT is a method for treating cancer by killing the cancer cells through generating lethal reactive oxygen species (ROS) near or within them. ROS are produced when a photosensitive molecule, called a photosensitizer, absorbs light of a specific wavelength, becomes electronically excited and reacts with dissolved oxygen in aqueous biological fluids. Since the penetration of illuminating light inside a tissue is again a concern, PDT in its standard form is not very effective unless the tumor is subcutaneous. Researchers found an improved PDT efficiency, as reflected in a substantially delayed tumor growth rate in live mice, when QD–RLuc was the source of light and Foscan was the photosensitizer. The QD, besides being the BRET acceptor with tunable emission and the central assembly platform for RLuc, played another more subtle role too. From earlier research, it appeared a shorter distance between the internal light source and the photosensitizer could improve the efficacy of PDT. The QD–Luc typically gets internalized via endocytosis that involves endosomes, as does the Foscan-loaded micelles used in this example. Therefore, the chances that the two encounter each other inside the same organelle are enhanced which in turn further improves PDT. The same may not happen with RLuc alone.

## 4. Developing New Designs

### 4.1. Anisotropic-QD–FLuc Constructs

**Figure 6:**
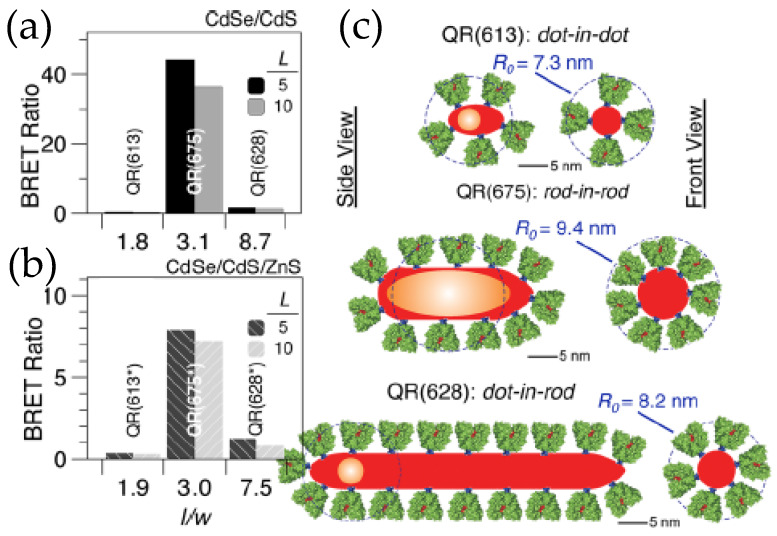
(**a**,**b**) BRET ratio of CdSe/CdS core/shell and CdSe/CdS/ZnS core/shell/shell quantum rods (QR), respectively, conjugated to the thermostable variant of firefly luciferase Photinus pyralis (Ppy) for two different Ppy:QR ratios of 5 and 10. (**c**) Schematic depicting the microstructure of various QRs used in the dot-in-dot configuration has the lowest aspect ratio while the dot-in-rod has the highest. While loading capacity increases in dot-in-rod configuration, the proteins that are too far from the core CdSe contribute very little to the actual BRET [55]. Copyright 2012 American Chemical Society.

The utility and fidelity of current QD–Luc configurations are certainly appreciated, but there is always room for improvements. The domain to do so could be the BRET efficiency to make QD–Luc probes look brighter or it could be the fabrication of a de novo functional nanodevice with the existing building blocks. For example, the QD–Luc constructs we discussed earlier involved exclusively spherical QDs that have a minimum surface area which restricts the number of luciferase that can be arrayed around them and which, in turn, limits the BRET ratio to only ~0.5–4.0. One of the ways researchers found to address this issue was by introducing quantum rods (QR) that have larger surface areas. In terms of photophysical properties, QRs are not very different from their spherical counterparts except that they display linearly polarized absorption and emission [53,54]. The polarization coupled with dimensionality and the internal microstructure of the rods have a dictating role in the efficiency of resonance energy transfer, which if carefully tuned can lead to a BRET ratio as high as 44. The QD’s high photoluminescent quantum yield contributes to the overall signal by increasing the QD’s light contribution at the portion of the spectrum where it emits. This will, in turn, help increase BRET ratio values. Maye’s lab worked on that facet by synthesizing a series of CdSe/CdS and CdSe/CdS/ZnS QRs with different aspect ratios and three different internal morphologies, a dot-in-dot, a dot-in-rod and a rod-in-rod, where core CdSe is spherical in the two former types and rod shaped in the latter (Figure 6) [55,56]. Their research unraveled the factors behind the dimensionality that actually controls the BRET ratio, which include not only the enzyme loading but also the distance between the donor and acceptor, and the polarization of the core. The distance between the donors, FLuc, and the acceptor, CdSe core, is effectively shorter in a rod-in-rod morphology. It is the QR with the same morphology that displays a strong polarization, which enhances the Förster distance (the distance at which FRET efficiency is 50%), and in turn improves the BRET efficiency. So their research suggests some guidelines that one needs to consider when designing BRET systems involving quantum confined semiconductor nanoparticles.

### 4.2. BRET-Multistep FRET Constructs

In parallel to the development of optimized BRET efficiency, the sophistication of such systems has steadily grown by integrating other elements, such as organic fluorophores [57] or FPs [58] to the existing constructs. They can work in concert with the QD to harvest luciferase light energy and utilize it by spatially and spectrally propagating it in a unique fashion that can potentially broaden their breadth of application. As a proof of concept, our group demonstrated that BRET between Luc and QD could be further coupled with multistep FRET, via which excitonic energy would cascade down a slanted energy landscape to redder region of the spectrum [57]. Technically this was achieved by concentrically arraying multiple copies of two different fluorescent dye labeled peptides, which, being tagged with His_6_, readily self-assembled onto the CdSe/ZnS core-shell QDs in a ratiometric manner. The fluorescent dyes, Alexa Fluor 647 and Cy5.5 acted as acceptors in the sequential FRET steps, the efficiency of which could be tuned, to some extent, by adjusting the dye to QD ratio.

**Figure 7:**
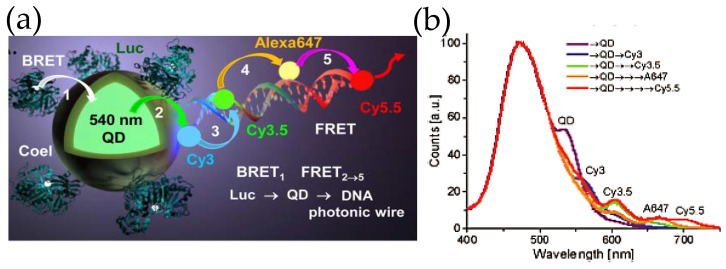
(**a**) Schematic depicting a self-assembled DNA photonic wire built onto QD–Luc conjugates. (**b**) The photonic wire, which was synthesized by hybridizing small fragments of DNA with pendant fluorescent organic dyes allows spatial and spectral propagation of excitonic energy via sequential FRET as displayed in the plot. The ratiometric self-assembly of Luc and the DNA wire, that relies upon His_6_-Zn^2+^ coordination chemistry, provides control over energy transfer efficiency. Reprinted with permission from [59]. Copyright 2015 American Chemical Society.

Inspired by the ability to sensitize multiple FRET acceptors within such constructs, our group took a step forward by attaching fluorescent dye-decorated DNA wires to QD–Luc hybrids (Figure 7) [59]. The DNA wire was fabricated by self-assembling multiple short pieces of DNA that were covalently tethered to different fluorescent dyes. The fluorescent dyes were chosen judiciously so that they display significant spectral overlap with the adjacent dye, which allowed the linearly arrayed dye ensemble to act as a photonic wire—transporting optical energy from one end to the other via sequential FRET. It was indeed noticed that Luc8 emission centered around ca. 480 nm could be transported to a spectrally and spatially distant dye Cy5.5 (λmax, em ~ 670 nm) via an array of fluorophores which besides QD540 included Cy3, Cy3.5 and AF647 fluorescent dyes. As far as sophistication is concerned, DNA photonic wires fall on the simpler side of the vast number of DNA based architectures that have already been synthesized and can potentially be given virtually any conceivable shape [60]. DNA as a nano-construction material not only allows precise arrangement of fluorophore, protein, antibody, or nanoparticle, [61] the relative distance and orientation of such elements can be modulated by external stimuli, like pH [62] or target DNA strand [63]. Smart designs unifying these elements with QD and luciferase could potentially lead to complex de novo photonic nanomaterials that could recognize a target, activate itself, process the light energy as necessary, and give us easily a readable signal both in vitro and in vivo, all in a standalone modality.

## 5. Conclusions and Outlook

Clearly, exploiting enzymatic-based BRET with semiconductor QDs as acceptors brings with it several unique photophysical and chemical benefits which, if properly developed, could lead to new, unique, and perhaps even translational bioapplications. In terms of just pure FRET-type donor-acceptor optical characteristics, the QD’s broad absorption profile, which increases nearly linearly from its first absorption band, allows the QD acceptor to be very effectively sensitized by the broad and blue-shifted emission from donor Luc substrates. The typical QD’s extinction coefficient also increases dramatically in this portion of the spectrum to values that approach several million. Such a spectral separation also functions as an effective Stokes shift, but more in terms of separating donor and acceptor emissions, which serves to simplify detection and deconvolution of their respective PL. This is especially true in the case of redder, near-IR emitting QDs, for example, those with 650–800 nm centered PL profiles. Moreover, the ability to array multiple discrete Luc enzymes around a given QD acceptor in a somewhat centrosymmetrical manner increases the probability that its dipole will couple to that of the excited-state substrate and engage in resonance energy transfer. The latter, however, does come at the expense of increasing the donor emission and consuming more substrate in a quicker manner as the number of enzymes per QD effectively increases. Nevertheless, the ability to accomplish this in a controllable manner represents, for all intents and purposes, a viable compromise between the desired benefit and any potential liability. Along with these properties comes the unique ability to multiplex and sensitize multiple QD acceptors via Luc in a localized and controlled spatiotemporal manner, as so amply demonstrated by Rao [26].

Perhaps the most unique and underexploited property of Luc–QD BRET-based sensitization is the ability to bypass the need for an external illumination source and directly allow for localized and controlled energy transfer. This is absolutely critical to using QDs as acceptors, since their broad absorption coupled to their long lifetimes and high quantum yields will cumulatively result in them being far better excited than any potential dye or FP donor they may be paired with; this confounding situation is not something that Förster most likely envisioned in his seminal derivation of the formalism that describes and accompanies this type of energy transfer. Beyond significantly reducing the background autofluorescence in a sample and any direct excitation of the QD acceptor, it actually gives the user almost absolute control over where and when the excitation takes place through the ability to add (or not) the chemical substrate and any accompanying cofactors. Interestingly, this is not something that has been actively utilized or exploited in any of the QD–Luc construct sensors and probes developed and demonstrated to date, beyond those that have been applied in vivo [26]. As to the further development of in vivo utility, be it for imaging or diagnostics, there is certainly much potential. However, this has to be tempered with first building a full understanding of QD toxicity in vivo. Although much work has been done to date on cellular toxicity of QDs [22,64,65,66], it certainly does not provide the necessary detail needed for in vivo use nor a full appreciation of the cost–benefit analysis that will form the basis for approved use as determined by regulatory agencies.

So where can we anticipate such QD constructs and the processes they exploit will evolve towards? Beyond a next generation of the imaging, probe, and sensor formats that are highlighted herein, one enticing prospect is that of creating stand-alone self-illuminating and light-harvesting/directing nanoscale constructs. The ability to couple these functionalities to the almost unlimited possibilities afforded by DNA nanotechnology at the nanoscale is particularly exciting [67,68]. This may allow the creation of a myriad of nanoscale disposable (smart) sensors that can be used in environmental, public health, medical, and point-of-care applications. Overall, this technology epitomizes what bionanotechnology, i.e., the intersection of biotechnology and chemistry/materials science, has to offer, and that is the creation of value-added or emergent materials and processes that can augment or drive development of new applications.

## Figures and Tables

**Table 1:** Intrinsic QD physicochemical properties that augment their role as bioluminescence resonance energy transfer (BRET) acceptors.

QD Acceptor Property	Effect on BRET
Non-trivial size with large surface-to-volume (s/v) ratio.	Allows for multiple luciferases to be displayed around the QD. ^1^ Allows for display of other biologicals on the QD surface.
Display of multiple Luc around the QD.	Increases the probability that BRET will occur.
Absorption increases to the blue.	Large spectral overlap with Luc emission.
Long excited lifetime, high quantum yield.	Bright QD acceptor PL. QD can act as donor or FRET relay to ternary or downstream acceptors.
Size-tunable PL.	Choice of QD PL emission window with large spectral separation from Luc emission.
Resistance to photo- and chemical degradation.	Allows for long-term robust use.
Can be surface functionalized with many different ligands	Provides access to different bioconjugation chemistries.

^1^ Such display is typically in a centrosymmetric manner.
